# Dystrophin R16/17-syntrophin PDZ fusion protein restores sarcolemmal nNOSμ

**DOI:** 10.1186/s13395-018-0182-x

**Published:** 2018-11-22

**Authors:** Aman Patel, Junling Zhao, Yongping Yue, Keqing Zhang, Dongsheng Duan, Yi Lai

**Affiliations:** 1 Department of Molecular Microbiology and Immunology, School of Medicine, University of Missouri, Medical Sciences Building, One Hospital Drive, Columbia, MO 65212 USA; 2 Department of Biomedical Sciences, College of Veterinary Medicine, University of Missouri, Columbia, MO 65212 USA; 3 Department of Neurology, School of Medicine, University of Missouri, Columbia, MO 65212 USA; 4 Department of Bioengineering, University of Missouri, Columbia, MO 65212 USA

**Keywords:** Neuronal nitric oxide synthase, Sarcolemma, Skeletal muscle, Dystrophin, Syntrophin, Fusion protein

## Abstract

**Background:**

Loss of sarcolemmal nNOSμ is a common manifestation in a wide variety of muscle diseases and contributes to the dysregulation of multiple muscle activities. Given the critical role sarcolemmal nNOSμ plays in muscle, restoration of sarcolemmal nNOSμ should be considered as an important therapeutic goal.

**Methods:**

nNOSμ is anchored to the sarcolemma by dystrophin spectrin-like repeats 16 and 17 (R16/17) and the syntrophin PDZ domain (Syn PDZ). To develop a strategy that can independently restore sarcolemmal nNOSμ, we engineered an R16/17-Syn PDZ fusion construct and tested whether this construct alone is sufficient to anchor nNOSμ to the sarcolemma in three different mouse models of Duchenne muscular dystrophy (DMD).

**Results:**

Membrane-associated nNOSμ is completely lost in DMD. Adeno-associated virus (AAV)-mediated delivery of the R16/17-Syn PDZ fusion construct successfully restored sarcolemmal nNOSμ in all three models. Further, nNOS restoration was independent of the dystrophin-associated protein complex.

**Conclusions:**

Our results suggest that the R16/17-Syn PDZ fusion construct is sufficient to restore sarcolemmal nNOSμ in the dystrophin-null muscle.

**Electronic supplementary material:**

The online version of this article (10.1186/s13395-018-0182-x) contains supplementary material, which is available to authorized users.

## Background

Nitric oxide synthases (NOS) catalyze the production of the signaling messenger, nitric oxide (NO). Neuronal NOS (nNOS) is the primary NOS isoform in skeletal muscle [1, 2]. nNOSμ is the primary nNOS isoform in muscle and it is localized at the sarcolemma. Sarcolemmal nNOSμ plays an important role in regulating multifaceted activities of muscle, including blood perfusion [3, 4], glucose metabolism [5–8], oxidative stress [9, 10], muscle contractility [11, 12], muscle satellite cell activation and muscle repair [13–18], mitochondria biogenesis [19–22], muscle mass [23–26], and muscle fatigue [26–29].

Activation of nNOSμ is dependent on dimerization of nNOSμ proteins. Each monomer contains a PDZ domain, an oxygenase domain, a calmodulin-binding site, and a reductase domain [30]. The interactions of two oxygenase domains mediate nNOSμ dimer formation and enzymatic activation [31, 32]. Loss of sarcolemmal nNOSμ impairs multiple NO-mediated activities [3, 4, 23, 29, 33]. nNOSμ localization at the sarcolemma depends on dystrophin and α1-syntrophin [34, 35]. Previously, biochemistry and X-ray crystallography studies have revealed that the α1-syntrophin PDZ domain binds to the nNOSμ PDZ domain [36, 37]. Transgenic studies further confirmed that the interaction between α1-syntrophin and nNOSμ is crucial for nNOSμ localization at the sarcolemma [3, 34, 38]. However, α1-syntrophin alone is not sufficient to anchor nNOSμ to the sarcolemma [35]. We have previously shown that dystrophin spectrin-like repeats 16 and 17 (R16/17) are essential for sarcolemmal localization of nNOSμ [39, 40]. Multiple lines of evidence, including yeast two-hybrid assay, biochemical and molecular analysis, and structural modeling, suggest that dystrophin R16/17 directly interact with nNOSμ PDZ domain [39–43]. Dystrophin is a sub-sarcolemmal protein. It maintains sarcolemmal integrity during muscle contraction and organizes a group of transmembrane and cytosolic proteins (such as dystroglycans, sarcoglycans, sarcospan, syntrophins, dystrobrevin, and nNOSμ) into the dystrophin-associated protein complex (DAPC) at the sarcolemma [44, 45].

Loss and/or diminished expression of dystrophin or DAPC components leads to a variety of muscular dystrophies such as Duchenne muscular dystrophy (DMD), Becker muscular dystrophy (BMD), and several forms of recessive limb-girdle muscular dystrophies (LGMD). Loss of sarcolemmal nNOS is a common feature in these muscular dystrophies [1, 35, 46–49]. Absence or reduction of sarcolemmal nNOS has also been reported in non-DAPC-related muscular dystrophies (eg. LGMD2B, MDC1A) [29, 50], inflammatory myopathies [50], cachexia [51], myasthenia gravis [26], diabetes [52], and aging-related muscle atrophy [53]. Collectively, accumulated evidence suggests that loss of sarcolemmal nNOSμ is a common manifestation in a wide variety of muscle diseases. Importantly, the absence of sarcolemmal nNOSμ has been shown to either directly or indirectly contribute to the initiation and progression of these diseases. Given the critical role sarcolemmal nNOSμ plays in various muscle activities, restoration of sarcolemmal nNOSμ should be considered as an important therapeutic goal.

Since dystrophin R16/17 and the syntrophin PDZ domain are both required for nNOSμ localization at the sarcolemma [36, 40], we hypothesize that a fusion protein consisting of dystrophin R16/17 and the syntrophin PDZ domain can anchor nNOSμ to the sarcolemma. In this study, we tested our hypothesis by expressing a membrane-bound dystrophin R16/17-syntrophin PDZ fusion protein with adeno-associated virus (AAV) in dystrophin-null muscle. This approach successfully restored sarcolemmal nNOSμ.

## Methods

### Animal care and studies

All animal procedures were carried out in accordance with NIH guidelines, and all animal experiments were approved by the Animal Care and Use Committee (ACUC) of the University of Missouri. BL10 mice (stock #: 000666), *Mdx* (stock #: 001801), DBA/2 J-*mdx* (stock #: 013141), and heterozygous Cmah/*mdx* (stock #: 017929) mice were purchased from The Jackson Laboratory. Homozygous Cmah/*mdx* mice were generated by breeding heterozygous Cmah/*mdx* mice. Both male and female mice were used in this study. All the mice are maintained in a specific-pathogen-free animal care facility with access to food and water ad libitum.

### Construct design

**Fig. 1:**
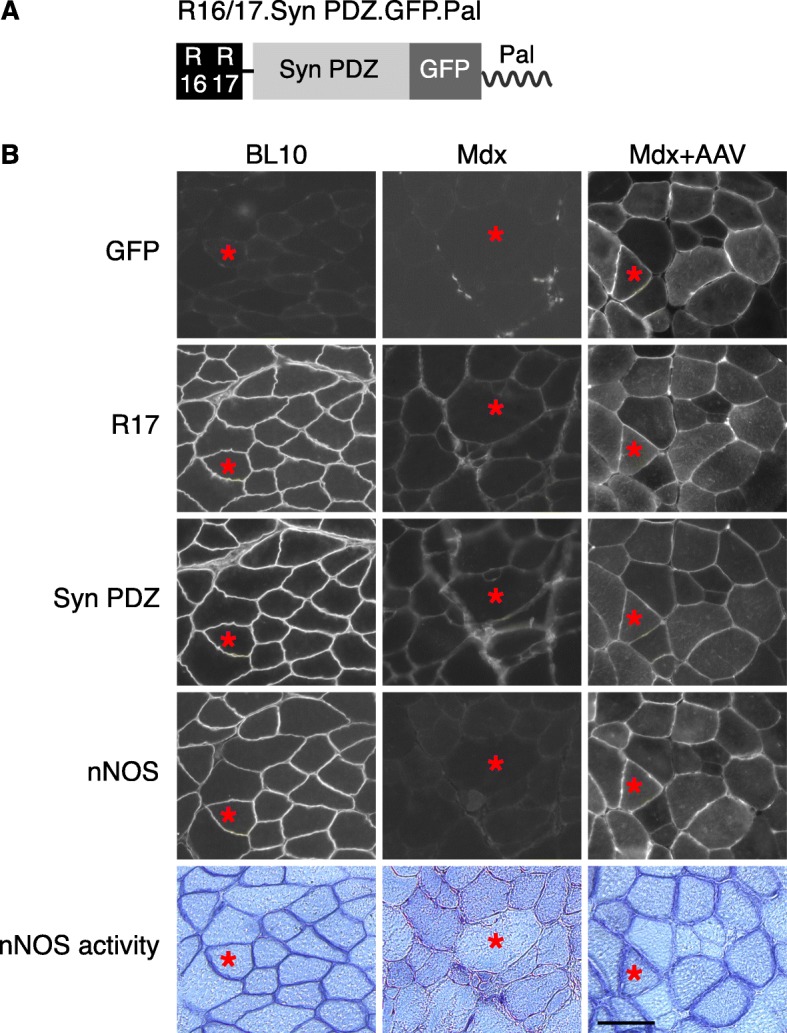
Dystrophin R16/17-syntrophin PDZ domain fusion protein restored sarcolemmal nNOSμ in *mdx* mice. **a.** Schematic outline of the fusion construct. R16/17, dystrophin spectrin-like repeats 16 and 17; Syn PDZ, syntrophin PDZ domain; Pal, the palmitoylation motif for membrane targeting. **b.** AAV viruses were injected into both TA muscles of six *mdx* mice (*n* = 6). Representative photomicrographs of serial muscle sections visualized for GFP, R17, syntrophin PDZ domain, and nNOS expression and nNOS activity from wild-type BL10 mice, untreated *mdx* mice, and AAV.R16/17-Syn PDZ.GFP.Pal treated *mdx* mice. Asterisk, the same myofiber in serial sections. Scale bar = 50 μm

In a previous study, we engineered an AAV construct (YL299) that carries the expression cassette of dystrophin R16/17.GFP with a membrane-targeting motif (Pal) [40]. The Pal motif is derived from the Ras palmitoylation sequence and has been successfully used to target nNOSμ, α-dystrobrevin-2a, and dystrophin R16/17 to the muscle membrane [40, 54, 55]. Here, we used YL299 as the backbone and inserted syntrophin PDZ domain between R17 and GFP. The linker sequence GGSG was included to connect dystrophin R16/17 and the syntrophin PDZ domain (Fig. 1a and Additional file 1: Figure S1). The syntrophin PDZ sequence was engineered into pYL299 by PCR-based cloning method using the full-length mouse syntrophin cDNA plasmid as the template (a gift from Dr. Stanley C. Froehner, University of Washington, Seattle, WA, USA). The resulting construct was named as YL465. In YL465, the expression of R16/17.Syn PDZ.GFP.Pal (Fig. 1a) was regulated by the CMV promoter and SV40 polyadenylation signal.

### AAV production and injection

Recombinant AAV-9 viruses were produced using our published protocol, which involves triple-plasmid transfection in the human embryonic kidney (HEK) 293 cells and three rounds of CsCl ultracentrifugation purification [40, 56]. AAV titer was determined by real-time PCR using Fast SYBR Green Master Mix kit (Applied Biosystems, Foster City, CA) with a pair of primers that amplify a fragment in the CMV promoter: forward primer: 5′-TTACGGTAAACTGCCCACTTG-3′; reverse primer: 5′-CATAAGGTCATGTACTGGGCATAA-3′.

A total of 5 × 10^11^ viral genome (vg) particles of AAV vectors (in a volume of 30 μl) were injected into the *tibialis anterior* (TA) muscle of six adult (2 to 4-month-old) *mdx*, three DBA/2 J-*mdx* [57–59], and three Cmah/*mdx* mice [60] according to our established method [40]. Four weeks after AAV injection, TA muscles were harvested, embedded in Tissue-Tek OCT (Sakura Finetek), and snap-frozen in 2-methylbutane with liquid nitrogen.

### Histology studies

Histology studies were performed on 10-μm cryosections of the TA muscles. General morphology of the muscle was examined by H.E. staining. GFP signal was detected by direct visualization under a fluorescence microscope. Dystrophin R17 domain was revealed by immunofluorescence staining with a mouse anti-R17 antibody (1:500, a gift from Dr. Glenn Morris, The Rober Jones and Agnes Hunt Orthopedic Hospital, Oswestry, Shropshire, United Kingdom). Sarcolemmal nNOSμ was identified by immunostaining with a rabbit anti-C-terminus of nNOS antibody (1:8000, N7280, Sigma), and nNOS activity staining was performed as we published before [56]. For nNOS activity staining, 16-μm cryosections were first fixed in 4% paraformaldehyde for 2 h at 4 °C. After a brief rinse in phosphate buffered saline (PBS), the tissue sections were permeabilized with 0.2% Triton X-100 at 37 °C for 20 min. The nicotinamide adenine dinucleotide phosphate (NADPH) diaphorase activity of nNOS was revealed by adding the mixture of 0.2% Triton X-100, 0.2 mM NADPH, and 0.16 mg/ml nitroblue tetrazolium (N6876-100MG, Sigma-Aldrich) [39, 40, 61]. nNOS activity appears as blue staining under the bright field. The syntrophin PDZ domain was detected by a mouse anti-pan-syntrophin antibody (1:500, ab11425, Abcam). β-dystroglycan was detected with a mouse anti-β-dystroglycan antibody (1:50, NCL-b-DG, Novocastra/Leica Biosystems). β-sarcoglycan was detected with a mouse anti-β-sarcoglycan antibody (1:50, NCL-b-SARC, Novocastra/Leica Biosystems). Dystrobrevin was detected with a mouse anti-dystrobrevin antibody (1:200, 610,766, BD Biosciences). At least three non-contiguous sections of each sample were examined by histology studies and representative images were present in the figures. In immunostaining, secondary antibody only was used as the negative control. Muscle histology was evaluated by two independent researchers, who were blinded for the information of experimental groups.

### Western blot

The whole TA muscle was homogenized by mechanical disruption with a mortar and a pestle in liquid nitrogen. Then, muscle tissues were lysed in the lysis buffer containing 10% sodium dodecyl sulfate (SDS), 5 mM ethylenediaminetetraacetic acid (EDTA), 62.5 mM Tris.HCl (pH 6.8), plus 1% cocktail proteinase inhibitor (11836153001, Roche Applied Science). The whole muscle lysate was obtained after spinning at 14,000 rpm for 2 min. The membrane-enriched microsomal fraction was extracted with the Plasma Membrane Protein Extraction kit (ab65400, Abcam) according to the manufacturer instructions [62]. The muscle lysates were separated on 6% or 8% SDS-polyacrylamide gel and transferred to the polyvinylidene difluoride (PVDF) membrane. The PVDF membranes were probed with the following antibodies: mouse anti-R17 antibody (1:500, a gift from Dr. Glenn Morris), mouse anti-pan-syntrophin antibody (1:600, ab11425, Abcam), rabbit anti-C-terminus of nNOS antibody (1:2000, N7280, Sigma), and rabbit anti-GFP antibody (1:1000, ab32146, Abcam). The protein loading was confirmed with an antibody against glyceraldehyde-3-phosphate dehydrogenase (GAPDH) (1:5000, MAB374, Millipore) for the whole lysate and an antibody against α1-Na^+^/K^+^ATPase (1:3000, ab7671, Abcam) for the microsomal fraction.

Quantification of immunoblotting was achieved by ImageStudioLite (Li-Cor Biosciences) according to the manufacturer’s instructions. Three different blots were quantified, and the signal of nNOS is normalized to that of GAPDH. The data were analyzed with the program GraphPad Prism 6.0a for Mac OS X (GraphPad Software, La Jolla, CA, USA). Comparison among three groups was done by ANOVA. Tukey’s test was used as the post hoc test to compare the difference between the two groups. The statistical significance was considered when the *P* value is less than 0.05.

## Results

### Membrane-bound R16/17-Syn PDZ fusion protein restored sarcolemmal nNOSμ in mdx muscle

We expressed the R16/17-Syn PDZ fusion protein (Fig. 1a) with AAV in *mdx* muscle. Expression of the fusion protein was characterized by the GFP signal and immunostaining with antibodies against R17 and the syntrophin PDZ domain. As shown in Fig. 1b, AAV delivery resulted in the efficient expression of the fusion protein at the sarcolemma in the dystrophic muscle. As revealed by positive nNOS immunostaining and activity staining at the sarcolemma, expression of the membrane-bound fusion protein successfully restored sarcolemmal nNOSμ in the dystrophin-null muscle (Fig. 1b).

**Fig. 2:**
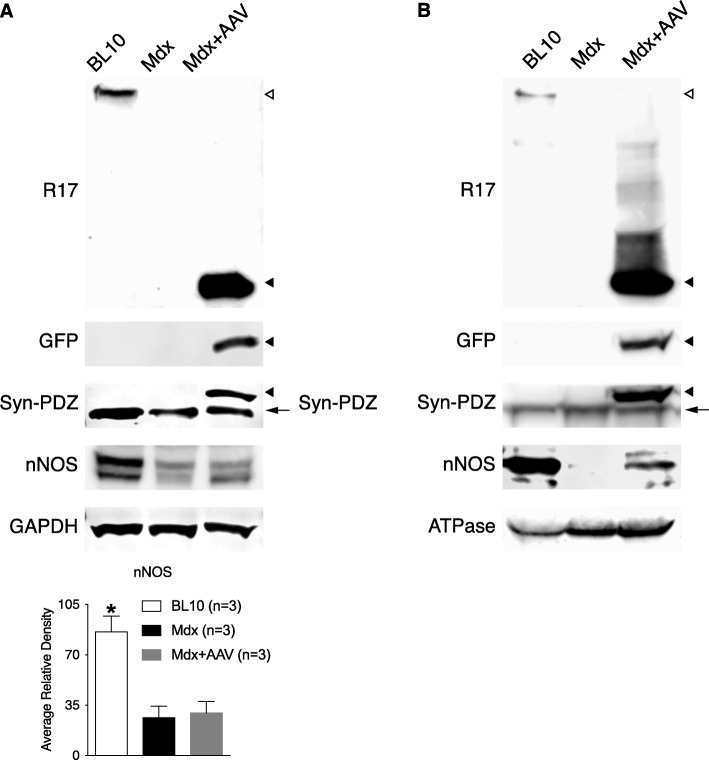
Immunoblot investigation of R16/17-Syn PDZ fusion protein expression and restoration of sarcolemmal nNOSμ in *mdx* mice. **a**. Whole muscle lysate western blot. The robust expression of the fusion protein was revealed by antibodies against R17, GFP, and the syntrophin PDZ domain. The total amount of nNOS in the AAV-injected muscle is almost the same as that of uninjected *mdx* muscle. Quantification of band intensity confirmed that the total amount of nNOS in the wild-type muscle is significantly higher than in *mdx* and AAV-injected muscles (asterisk, significantly different from *mdx* or AAV-injected muscle). GAPDH is the loading control. **b**. Microsomal preparation western blot. The membrane expression of the fusion protein was detected by antibodies against R17, GFP, and the syntrophin PDZ domain. Restoration of sarcolemmal nNOSμ by the fusion protein was confirmed by identifying nNOS in the microsomal preparation of AAV-injected muscle. ATPase is the loading control. Open arrowhead, full-length dystrophin; filled arrowhead, R16/17-Syn PDZ fusion protein; arrow, endogenous syntrophin

Western blot was performed to corroborate the expression of the fusion protein and restoration of sarcolemmal nNOSμ. The robust expression of the fusion protein was confirmed by immunoblotting with antibodies against R17, GFP, and the syntrophin PDZ domain in the whole muscle lysate (Fig. 2a). Consistent with previous studies [1, 12], dystrophin deficiency significantly reduced the total amount of nNOSμ in the muscle. AAV-mediated expression of the R16/17-Syn PDZ did not change the total nNOSμ level in *mdx* muscle (Fig. 2a). Membrane localization of the fusion protein was validated by microsomal preparation western blot with antibodies against R17, GFP, and the syntrophin PDZ domain (Fig. 2b). Importantly, restoration of sarcolemmal nNOSμ was confirmed by identifying nNOSμ in the microsomal fraction of the AAV-injected *mdx* muscle (Fig. 2b).

### Restoration of sarcolemmal nNOSμ by the fusion protein was independent of the DAPC

**Fig. 3:**
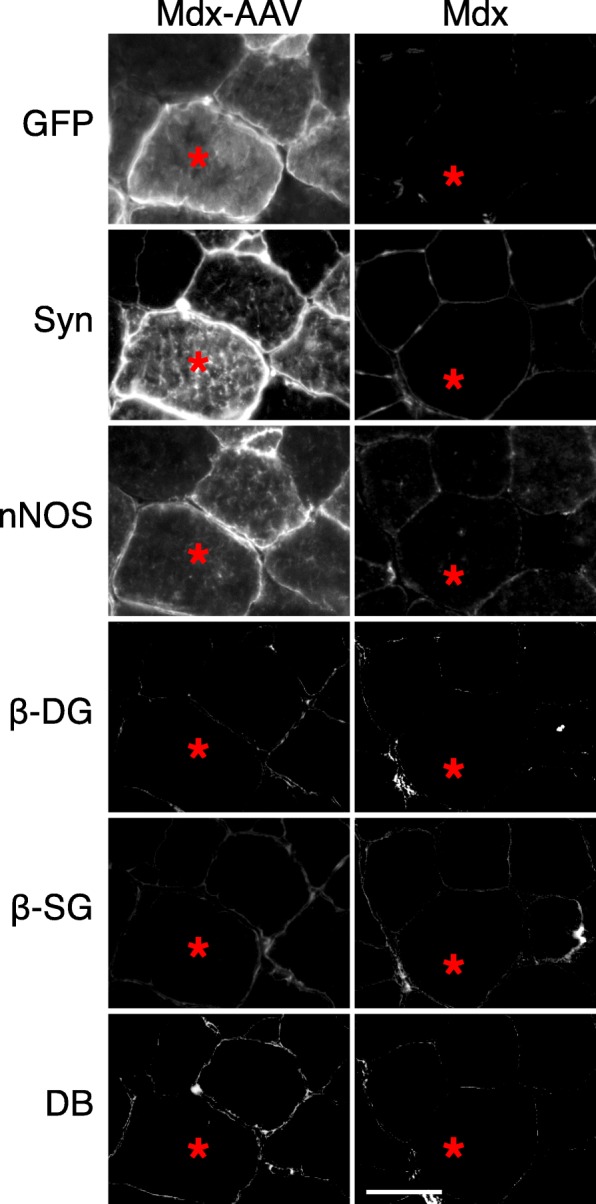
Restoration of sarcolemmal nNOSμ by the R16/17-Syn PDZ fusion protein was independent of the DAPC. Representative photomicrographs of serial muscle sections visualized for GFP, syntrophin PDZ domain, nNOS expression, β-dystroglycan (β-DG), β-sarcoglycan (β-SG), and dystrobrevin (DB) in uninjected *mdx* mice and AAV.R16/17-Syn PDZ.GFP.Pal treated *mdx* mice. Asterisk, the same myofiber in serial sections. Scale bar = 50 μm

Next, we analyzed whether restoration of sarcolemmal nNOSμ by the R16/17-Syn PDZ fusion protein requires the presence of other DAPC components. As reported previously [63, 64], sarcolemmal expression of β-dystroglycan, β-sarcoglycan, dystrobrevin, syntrophins, and nNOS was greatly reduced in uninjected *mdx* muscle (Fig. 3). In the serial sections of AAV-injected muscle, GFP signal, syntrophin staining, and nNOS staining showed sarcolemmal expression of the fusion protein and restoration of nNOSμ sarcolemmal localization. However, immunostaining for β-dystroglycan, β-sarcoglycan, and dystrobrevin showed similar levels of expression as that of uninjected *mdx* muscle (Fig. 3).

### The fusion protein also restored sarcolemmal nNOSμ in Cmah/mdx and DBA/2 J-mdx mice

**Fig. 4:**
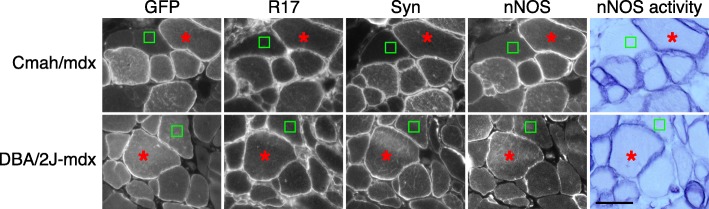
The R16/17-Syn PDZ fusion protein restored sarcolemmal nNOSμ in Cmah/*mdx* and DBA/2 J-*mdx* mice. AAV viruses were injected into both TA muscles of three Cmah/*mdx* (*n* = 3) and three DBA/2 J-*mdx* mice (*n* = 3). Representative photomicrographs of serial muscle sections visualized for GFP, R17, syntrophin (Syn), nNOS expression, and nNOS activity staining. In non-transduced myofibers, there is no sarcolemmal nNOSμ restoration. Asterisk, the same myofiber in serial sections; square, the non-transduced myofiber. Scale bar = 50 μm

Cmah/*mdx* and DBA/2 J-*mdx* mice are two severe mouse DMD models [57–60]. In addition to the mutation in the dystrophin gene, Cmah/*mdx* mice also carry an inactivating deletion in the CMAH (cytidine monophosphate sialic acid hydroxylase) gene [60]. DBA/2 J-*mdx* congenic mice were generated by backcrossing C57BL/10-*mdx* with DBA-2 J inbred mice for several generations. To test whether our fusion construct can restore sarcolemmal nNOSμ localization in mice with more severe phenotypes, we performed AAV injection in Cmah/*mdx* and DBA/2 J-*mdx* mice. Similar to that of *mdx* mice **(**Figs. 1 and 3**)**, AAV injection resulted in the expression of the R16/17-Syn PDZ fusion protein at the sarcolemma (Fig. 4). nNOS immunostaining and activity staining showed the recovery of sarcolemmal nNOSμ in serial muscle sections (Fig. 4).

## Discussion

In this study, we engineered a fusion protein consisting of the minimal components required for sarcolemmal nNOSμ localization and expressed the fusion protein with AAV gene transfer in dystrophin-null mice. Microscopic examination revealed successful expression of the fusion protein at the sarcolemma and restoration of membrane-bound nNOSμ (Figs. 1, 2, and 4). These findings were confirmed by whole muscle lysate and microsomal preparation western blot (Figs. 1 and 2). We also showed that restoration of sarcolemmal nNOSμ by the fusion protein was independent of other components of the DAPC (Fig. 3).

In our previous studies, we demonstrated that dystrophin R16/17 serves as the nNOS-binding domain in the context of a dystrophin protein [39, 40]. We tested a series of R16/17-containing mini- and micro-dystrophins. These synthetic R16/17-inclusive dystrophins successfully restore sarcolemmal nNOSμ expression in the murine and canine DMD models and offer better protection than those without R16/17 in animal models [39, 65–70]. An R16/17-inclusive micro-dystrophin has already been used in a clinical trial [71]. However, it remained elusive whether R16/17 alone can restore sarcolemmal nNOSμ in the dystrophin-null muscle. We have previously tested AAV constructs that expressed dystrophin R16/17 with and without Pal motif. Without Pal motif, R16/17 are confined to the cytosol of myofibers, while with Pal motif, R16/17 are targeted to the muscle membrane [40]. In *mdx* mice, membrane-bound R16/17 did not restore sarcolemmal nNOSμ. However, the exact same construct successfully restored sarcolemmal nNOSμ in ΔH2-R19 mini-dystrophin transgenic mice [40]. R16/17 are missing in both *mdx* mice and ΔH2-R19 mini-dystrophin transgenic mice. Most sarcolemmal DAPC components (dystroglycans, sarcoglycans, syntrophin, and dystrobrevin but not nNOS) were restored in transgenic mice but not *mdx* mice [63, 64, 72, 73]. Since syntrophin has been shown to interact with nNOSμ [36, 37], we reasoned that successful restoration of sarcolemmal nNOSμ in transgenic mice was likely due to the presence of syntrophin at the sarcolemma in transgenic mice. Similarly, we speculated that failure to restore sarcolemmal nNOSμ in *mdx* mice by membrane-bound R16/17 was due to the lack of sarcolemmal syntrophin [63, 64, 72, 73]. However, several important issues remain unclear. First, it is not clear whether other DAPC components (dystroglycans, sarcoglycans, and dystrobrevin) have contributed to the successful restoration of sarcolemmal nNOSμ in transgenic mice. Second, since dystrophic phenotype was largely attenuated in transgenic mice, it is unclear whether the improved muscle microenvironment also plays a role. To address these questions, we now generated the membrane-bound R16/17-Syn PDZ construct and tested it in mildly affected *mdx* mice and in Cmah/*mdx* and DBA/2 J-*mdx* mice, two severely affected DMD mouse models. Although the membrane-bound R16/17-Syn PDZ construct did not restore other DAPC components (such as β-dystroglycan, β-sarcoglycan, and dystrobrevin) (Fig. 3), nor was the muscle disease attenuated, the fusion construct successfully restored sarcolemmal nNOSμ. These results suggest that R16/17 and the syntrophin PDZ domain are the only components required for the sarcolemmal localization of nNOSμ. Other DAPC components and muscle microenvironment have a nominal impact.

**Fig. 5:**
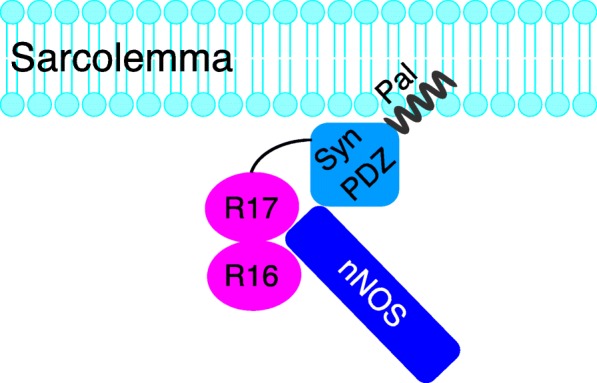
The model of sarcolemmal nNOS restoration by R16/17-Syn PDZ fusion protein. The R16/17-Syn PDZ fusion protein is targeted to the sarcolemma by the membrane-targeting motif (Pal). R16/17 and the syntrophin PDZ domain in the fusion protein interact with nNOSμ PDZ domain to anchor nNOSμ to the sarcolemma (not drawn to scale). Please note an activated nNOSμ exists as a dimer. It is currently unclear how exactly the two PDZ domains in the nNOSμ dimer interact with dystrophin R16/17 and the syntrophin PDZ domain. It is possible that one nNOSμ PDZ domain interacts with dystrophin R16/17 and the other nNOSμ PDZ domain interacts with the syntrophin PDZ domain. It is also possible that a single nNOSμ PDZ domain can interact with both dystrophin R16/17 and the syntrophin PDZ domain. For simplicity, the activated nNOSμ dimer was depicted as a single molecule in this cartoon

In all the published studies, R16/17 and syntrophin are provided separately as two independent molecules. From a drug development standpoint, this is less appealing. Ideally, one would want to put both R16/17 and the syntrophin PDZ domain together as one molecule. However, putting R16/17 next to the syntrophin PDZ domain in a single molecule may create a spatial hindrance for the simultaneous interaction of nNOSμ with both R16/17 and the syntrophin PDZ domain. Our results suggest that this is not an issue (Fig. 5). Dimerization of nNOSμ is essential for its activity. Although both R16/17 and syntrophin PDZ directly interact with nNOSμ PDZ domain, it remains to be determined whether the single PDZ domain of a nNOSμ monomer bind to both dystrophin R16/17 and syntrophin PDZ domain, or two different PDZ domains of an activated nNOS dimer interact with R16/17 and syntrophin PDZ separately, namely one nNOSμ PDZ domain interacts with R16/17 and the other nNOSμ PDZ domain interacts with syntrophin (Fig. 5).

Our study has several limitations. First, we have only limited our investigation in mouse DMD models. Future studies in other disease models (such as cachexia and aging-related muscle atrophy, inflammatory myopathies, myasthenia gravis, and other muscular dystrophies that display sarcolemmal nNOSμ delocalization) will be helpful to determine whether our fusion construct can serve as a universal treatment to restore membrane-associated nNOSμ expression in these conditions. Second, our current study has mainly focused on the morphological and biochemical demonstration of sarcolemmal nNOSμ restoration. Future functional and physiological studies are needed to show improvements in nNOSμ regulated muscle activities and signaling pathways with the dystrophin R16/17-syntrophin PDZ fusion protein.

## Conclusions

In this study, we found that the fusion protein containing dystrophin R16/17 and syntrophin PDZ domain restored sarcolemmal nNOSμ in dystrophin-null mice. Sarcolemmal nNOSμ restoration by the fusion protein is independent of the DAPC. This fusion construct has established the basis for developing a universal treatment to restore sarcolemmal nNOSμ in a wide variety of muscle diseases.

## Additional file

Additional file 1: **Figure S1.** The full amino acid sequence of dystrophin R16/17-syntrophin PDZ.GFP.Pal fusion protein. The subdomains of the fusion protein was annotated by different colors (R16, R17, linker, syntrophin-PDZ, GFP, Pal). (PDF 56 kb)

## Abbreviations

AAV: Adeno-associated virus
ACUC: Animal care and use committee
ANOVA: Analysis of variance
BMD: Becker muscular dystrophy
CMAH: Cytidine monophosphate sialic acid hydroxylase
CMV: Cytomegalovirus
DAPC: Dystrophin-associated protein complex
DMD: Duchenne muscular dystrophy
EDTA: Ethylenediaminetetraacetic acid
GAPDH: Glyceraldehyde-3-phosphate dehydrogenase
HEK: Human embryonic kidney
LGMD: Limb-girdle muscular dystrophy
MDC1A: Merosin-deficient congenital muscular dystrophy
NADPH: Nicotinamide adenine dinucleotide phosphate
nNOS: Neuronal nitric oxide synthase
NO: Nitric oxide
PBS: Phosphate buffered saline
PVDF: Polyvinylidene difluoride
SDS: Sodium dodecyl sulfate
TA: Tibialis anterior
