# Supplementary material for: Dystrophin R16/17-syntrophin PDZ fusion protein restores sarcolemmal nNOSμ
Source: Skelet Muscle. 2018 Nov 22;8:36. doi: 10.1186/s13395-018-0182-x (PMC6251231; doi:10.1186/s13395-018-0182-x)
Supplement: Supplementary file 1 — Figure S1. The full amino acid sequence of dystrophin R16/17-syntrophin PDZ.GFP.Pal fusion protein. The subdomains of the fusion protein was annotated by different colors (R16, R17, linker, syntrophin-PDZ, GFP, Pal). (PDF 56 kb) [file 13395_2018_182_MOESM1_ESM.pdf]

Human Dys R16

MEISYVPSTYLTEITHVSQALLEVEQLLNAPDLCAKDFEDLFKQEESLKNIKDSLQQSSGRIDIIHSKKTAALQSATPVER

Human Dys R17

VKLQEALSQLDFQWEKVNKMYKDRQGRFDRSVEKWRRFHYDIKIFNQWLTEAEQFLRKTQIPENWEHAKYKWYLKELQDGI

Linker

GQRQTVVRTLNATGEEIIQQSSKTDASILQEKLGSLNLRWQEVCKQLSDRKKRLEEGGSGLLLQRRRVTVRKADAGGLGIS

Mouse α1-syntrophin PDZ

IKGGRENKMPILISKIFKGLAADQTEALFVGDAILSVNGEDLSSATHDEAVQALKKTGKEVVLEVKYMKEVSPYFILEVSK

GFP

GEELFTGVVPILVELDGDVNGHKFSVSGEGEGDATYGKLTLKFICTTGKLPVPWPTLVTTLTYGVQCFSRYPDHMKQHDFF

KSAMPEGYVQERTIFFKDDGNYKTRAEVKFEGDTLVNRIELKGIDFKEDGNILGHKLEYNYNSHNVYIMADKQKNGIKVNF

Pal

KIRHNIEDGSVQLADHYQQNTPIGDGPVLLPDNHYLSTQSALSKDPNEKRDHMVLLEFVTAAGITLGMDELYKKDGKKKKK

KSKTKCVIM

Supplementary Fig 1. The full amino acid sequence of dystrophin R16/17-syntrophin PDZ.GFP.Pal fusion protein. The subdomains of the fusion protein was annotated by different colors (R16, R17, linker, syntrophin-PDZ, GFP, Pal).
